# Decoding the triglyceride-glucose index in metabolic dysfunction-associated steatotic liver disease: integrative insights from Mendelian randomization, cross-tissue transcriptomics, and spatial multi-omics

**DOI:** 10.1097/JS9.0000000000003576

**Published:** 2025-10-07

**Authors:** Shuxu Wei, Lingbin He, Youti Zhang, Xinyi Li, Suiqin Zhong, Ling Xiao, Ronghuai Shen, Xiaojia Lu, Zhouwu Shu, Yan Quan, Xianxi Huang

**Affiliations:** a Department of Cardiology, The First Affiliated Hospital of Shantou University Medical College, Shantou, China; b Laboratory of Molecular Cardiology, The First Affiliated Hospital of Shantou University Medical College, Shantou, China; c Department of Cardiology, Jiexi People’s Hospital, Jieyang, Guangdong, China; d Shan-Jie Joint Research Institute for Chronic Diseases (SJRICD), Jiexi people‘s hospital, Jieyang, Guangdong, China; e Department of Surgery, The First Affiliated Hospital of Shantou University Medical College, Shantou, China

**Keywords:** gene expression profiling, Mendelian randomization, metabolic dysfunction-associated steatotic liver disease, single-cell spatial transcriptomics, triglyceride-glucose index

## Abstract

**Background:**

The triglyceride-glucose (TyG) index, an insulin resistance marker linked to the progression of metabolic dysfunction-associated steatotic liver disease (MASLD), underscores the redox imbalance-mediated crosstalk between MASLD and cardiovascular-liver-metabolic health (CLMH), although its causal mechanisms and molecular drivers remain unresolved.

**Methods:**

We employed a multi-omics framework to integrate Mendelian randomization (MR) and transcriptome-wide association studies (TWAS). MR leveraged 192 genome-wide significant single-nucleotide polymorphisms for TyG from the UK Biobank, employing inverse-variance weighted (IVW) and generalized summary-data MR (GSMR). Transcriptomic integration utilized four approaches: Multi-marker Analysis of GenoMic Annotation for gene-set enrichment; Joint-Tissue Imputation PrediXcan (JTI-PrediXcan) for tissue-specific expression; Sparse Multi-Tissue Imputation Xcan (SMulTiXcan) for cross-tissue meta-analysis; and Fine-mapping of Causal Gene Sets (FOCUS) for Bayesian fine-mapping. Comorbid genes were validated using Functional Summary-based Imputation (FUSION) and prioritized based on the Polygenic Priority Score (PoPS). Single-cell spatial transcriptomics (sc-ST) in embryonic mice (E16.5) mapped tissue-specific expression via genetically informed spatial mapping (gsMap).

**Results:**

The MR analysis demonstrated a causal effect of TyG on MASLD risk [IVW: odds ratio (OR) = 1.58, 95% CI = 1.04–2.38, *P* = 0.030; GSMR: OR = 1.43, 95% CI = 1.27–1.61, *P* = 5.20 × 10^−9^]. TWAS identified 12 comorbid genes (C2orf16/SPATA31H1, FNDC4, GCKR, GMIP, HAPLN4, LPAR2, MAU2, MEF2B, NDUFA13, NRBP1, TM6SF2, and ZNF513). Independent validation using the FUSION framework confirmed nine TyG-MASLD comorbid genes with genome-wide significant false discovery rate-adjusted associations. Notably, TM6SF2 (TyG-PoPS = 7.2491) and GCKR (TyG-PoPS = 6.7102) showed strong positive associations in TyG, whereas NDUFA13 exhibited negative scores in MASLD (PoPS = −0.5028). Spatial mapping revealed conserved enrichment of APOA1, APOB, and APOC4 (sc-ST, *P* < 0.001) in murine liver and vascular tissues. Organ-specific analysis showed significant MASLD signals including the liver (sc-ST, *P* = 6.43 × 10^−5^), adrenal gland (Cauchy *P* = 0.0064), and connective tissue (sc-ST, *P* = 3.29 × 10^−5^).

**Conclusion:**

This study establishes TyG as a causal MASLD driver mediated by redox-sensitive hubs and evolutionarily conserved apolipoproteins, linking hepatic lipid peroxidation to systemic metabolic dysregulation. Targeting these pathways may mitigate dual hepatic-cardiovascular risks, advancing precision therapies for CLMH.

**HIGHLIGHTS**

- Causal TyG-MASLD link confirmed via MR (OR=1.58).
- 12 comorbid genes (e.g., TM6SF2, GCKR) bridge metabolic-cardiovascular pathways.
- Cross-species spatial mapping reveals conserved lipid genes (APOA1, APOB, APOC4).
- MASLD exhibits broader methodological concordance than TyG in transcriptomics.
- Multi-omics framework integrates MR, TWAS, and gsMap for CLMH insights.

## Introduction

Metabolic dysfunction-associated steatotic liver disease (MASLD) represents a chronic liver condition closely linked to systemic metabolic abnormalities^[1]^. Its diagnostic criteria have evolved from the exclusion-based “nonalcoholic fatty liver disease” (NAFLD)^[2,3]^ to a positive framework requiring hepatic steatosis (confirmed by imaging or biopsy) and at least one cardiometabolic risk factor, better capturing the multisystem pathophysiology of the disease^[1,4]^. Among those with type-2 diabetes, prevalence rises to 58.84% in East Asian and 72.65% in Western populations. Sedentary lifestyles and dietary shifts away from traditional patterns towards high-fat/sugar intake contribute to this by promoting gut dysbiosis^[5]^. Although often asymptomatic and incidentally detected through abnormal liver tests or imaging, MASLD carries significant risks of progression to metabolic dysfunction-associated steatohepatitis (MASH) and fibrosis, alongside increased hepatocellular carcinoma incidence in early-stage disease and gallstone formation from supersaturated bile^[6]^. Hepatolithiasis risk is notably elevated in patients with biliary tract malformations^[7]^. Cardiovascular disease (CVD) is the main cause of death in MASLD. Hepatic fat, insulin resistance, endothelial dysfunction, and systemic inflammation drive this risk, leading to accelerated atherosclerosis, diastolic dysfunction, and arrhythmias^[8,9]^.

Current MASLD management focuses on lifestyle changes: Mediterranean diet and ≥150 min/week moderate exercise. Weight loss of ≥3–5% improves steatosis and ≥10% reverses MASH/fibrosis^[4]^. Glucagon-like peptide-1 receptor agonists and sodium-glucose cotransporter-2 inhibitors offer triple benefits: glycemic control, weight loss, and cardiovascular protection^[10,11]^. Emerging agents, including peroxisome proliferator-activated receptor (PPAR) agonists and thyroid hormone receptor-β agonists, further show histological improvements in MASH^[2,12]^. However, clinical challenges persist: specific, noninvasive methods are still lacking to detect mild steatosis and to accurately identify at-risk groups using tools like the Fibrosis-4 index or abdominal ultrasound^[13]^. Conventional cardiovascular risk models tend to underestimate MASLD as an independent risk factor^[9]^. Additionally, gaps persist in clinician awareness and interdisciplinary care coordination^[14]^. Furthermore, the mechanistic heterogeneity and variable therapeutic responses observed in lean MASLD patients warrant further investigation^[15]^.

The triglyceride-glucose index (TyG) serves as a simple yet robust marker of insulin resistance^[16,17]^. Elevated TyG correlates with symptomatic coronary artery disease severity and predicts major adverse cardiovascular events in spatial transcriptomics (ST) elevated myocardial infarction patients^[18]^. It also associates with arterial stiffness, renal microvascular damage, and incident diabetes, highlighting its utility in multisystem metabolic risk assessment^[19,20]^. MASLD and TyG are driven by insulin resistance and dyslipidemia. Hepatic fat and systemic inflammation in MASLD promote endothelial dysfunction and atherosclerosis, raising CVD risk^[8,9]^. TyG, reflecting hepatic insulin resistance and lipotoxicity, may identify MASLD patients at heightened risk of steatosis progression and fibrosis^[14]^. Its sensitivity might improve risk stratification in populations that are underestimated by traditional models^[13,15]^.

Mendelian randomization (MR) is a key method for studying disease causes, especially when randomized controlled trials are not possible. It uses single-nucleotide polymorphisms (SNPs) strongly linked to exposures as instrumental variables (IVs) to mimic RCT randomization via the genetic lottery at conception^[21,22]^. This approach inherently minimizes confounding biases, as genetic variants remain unaffected by postnatal factors like age or sex^[23]^. Transcriptome-wide association studies (TWAS) integrate expression quantitative trait loci (eQTL) data with genome-wide association study (GWAS) summary statistics to identify candidate genes and investigate gene–trait associations^[24]^. Methods such as Multi-marker Analysis of GenoMic Annotation (MAGMA)^[25]^ and Functional Summary-based Imputation (FUSION)^[26]^ are employed.

The primary objective of this study is to investigate the association between the TyG index and MASLD through MR and genomic analyses, utilizing 192 strongly correlated SNPs^[27]^ as proxies for TyG. This approach aims to identify shared genetic pathways and advance integrated strategies for cardiovascular-liver-metabolic health (CLMH). The significance of this work lies in three key aspects. First, the TyG index may serve as a complementary tool for early MASLD screening by identifying individuals with insulin resistance and metabolic abnormalities, thereby improving the sensitivity of noninvasive diagnostic methods. Second, combining TyG with hepatic fat quantification could refine cardiovascular risk stratification in MASLD patients, enabling personalized management. Furthermore, longitudinal monitoring of TyG dynamics may evaluate the efficacy of lifestyle modifications or pharmacological interventions in improving metabolic and cardiovascular outcomes. In accordance with the TITAN Guidelines 2025 (Supplemental Digital Content Materials, available at: http://links.lww.com/JS9/F272) governing the declaration and use of artificial intelligence, we confirm that no AI tools were utilized in the data generation, analysis, or manuscript preparation of this study^[28]^.

## Methods

We initially collected 192 strongly associated proxy SNPs for the TyG index from a UK Biobank cohort study with genetic risk scores^[27]^, followed by imputation using the GRCh37 reference genome from the 1000 Genomes Project European population to generate chromosome positions, base pair locations, and effect allele frequencies (EAF). MR was applied to evaluate causal relationships between TyG and MASLD using these SNPs. Transcriptomic analyses integrated four complementary approaches: (1) MAGMA for gene-set enrichment analysis^[25]^; (2) Joint-Tissue Imputation Enhanced PrediXcan (JTI-PrediXcan) to predict tissue-specific gene expression^[29,30]^; (3) Summary-based Multi-Tissue Imputation Xcan (SMulTiXcan) to aggregate cross-tissue expression signals^[29,31]^; and (4) Fine-mapping of Causal Gene Sets (FOCUS) to prioritize causal genes via Bayesian fine-mapping^[32]^. Consensus genes were defined by concordance across two or more methods. FUSION was independently applied to validate shared gene–MASLD associations through expression-trait colocalization^[26]^. Candidate genes were further prioritized using the Polygenic Priority Score (PoPS), which integrates functional genomic annotations and pleiotropic evidence to rank genes with robust associations^[33]^. Finally, single-cell spatial transcriptomics (sc-ST) was integrated with MASLD GWAS data, leveraging embryonic mouse E16.5 ST datasets spanning 25 organs to systematically map MASLD-associated cellular architectures and organ-specific distributions of shared genes at single-cell resolution^[34]^.

**Figure 1.**
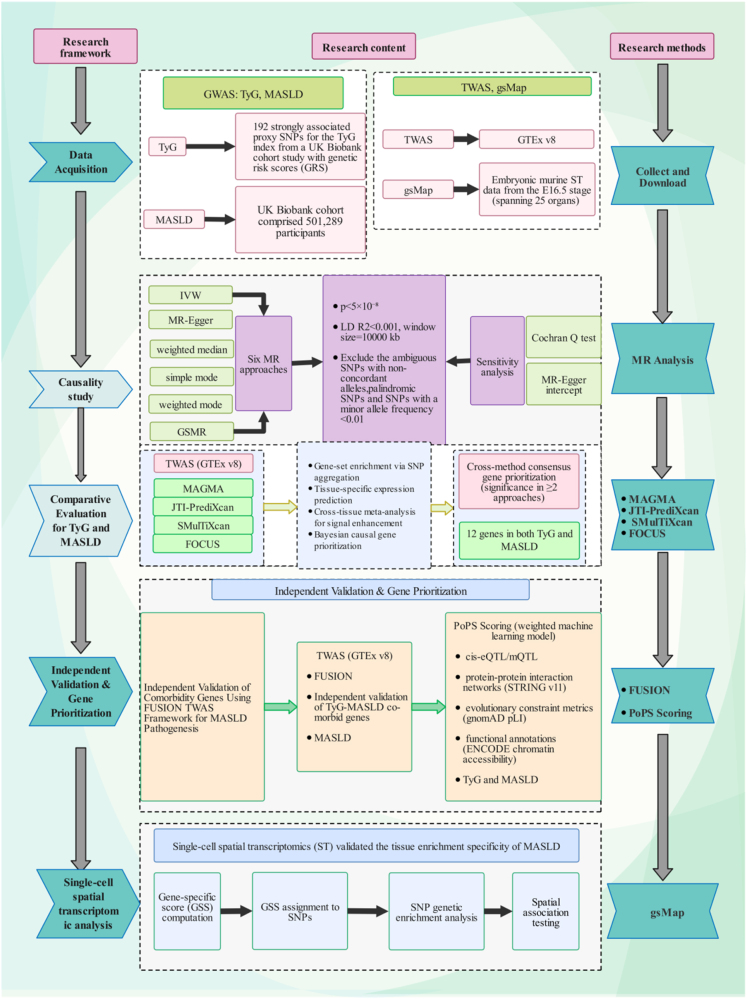
Flow chart of our study. GWAS, Genome-Wide Association Study; TyG, triglyceride-glucose index; MASLD, metabolic dysfunction-associated steatotic liver disease; SNPs, single nucleotide polymorphisms; GRS, genetic risk scores; TWAS, transcriptome-wide association study; GTEx, Genotype-Tissue Expression Project; gsMap, genetically informed spatial mapping; MR, Mendelian randomization; IVW, inverse variance weighted; GSMR, Generalized Summary-data Mendelian Randomization; LD, linkage disequilibrium; MAF, Minor Allele Frequency; MAGMA, Multi-marker Analysis of GenoMic Annotation; JTI-PrediXcan, Joint-Tissue Imputation PrediXcan; SMulTiXcan, Sparse Multi-Tissue Imputation Xcan; FOCUS, Fine-mapping of Causal Gene Sets; FUSION, Functional Summary-based Imputation; PoPS, Polygenic Priority Score; STRING, Search Tool for Recurring Instances of Neighboring Genes; gnomAD, Genome Aggregation Database; pLI, Probability of Loss-of-function Intolerance; ENCODE, Encyclopedia of DNA Elements; GSS, Gene-Specific Score.

All analyses utilized R (R-4.4.1) and Python, with detailed software and methodological specifications provided in Supplemental Digital Content Table S1, available at: http://links.lww.com/JS9/F463. The flow chart is shown in Figure 1. This study met the requirements of the STROCSS 2025 Guideline Checklist^[35]^.

### Sources of TyG and MASLD data

In the Methodology section about MASLD data analysis, we utilized the 2025 updated dataset derived from a comprehensive GWAS study employing UK Biobank resources^[36]^. This study cohort comprised 501 289 participants with documented self-reported sex and birth dates, enabling rigorous evaluation of verified MASLD case-control status. GWAS summary statistics for the TyG index were acquired from the UK Biobank, a population-based cohort comprising 273 368 European-ancestry participants aged 40–69 years, excluding individuals with pre-existing diabetes mellitus (DM) or lipid metabolism disorders^[27]^. IV selection followed stringent criteria: SNPs achieving genome-wide significance (*P* < 5 × 10^−8^) were identified via linear regression models adjusted for age, sex, and the top five genetic principal components to mitigate population stratification. Subsequent clumping (clump *R*^2^ < 0.01, 10 000kb window) and exclusion of SNPs associated with glucose (GLU) or triglyceride (TG) levels yielded 192 independent TyG-associated SNPs for downstream analyses. The above SNPs were interpolated using the human reference genome GRCH37 reference file from the European 1000 Genomes Project to generate chromosome and base pair location and EAF. The full list of 192 SNPs is provided in Supplemental Digital Content Table S2, available at: http://links.lww.com/JS9/F463. During GRS construction for TyG indices, individuals with diabetes mellitus and dysregulated lipoprotein metabolism were excluded. SNPs were stringently filtered, removing those associated with TG/GLU levels or correlated with non-lipid/non-glycemic traits: systolic blood pressure, diastolic blood pressure, and body mass index^[27]^. These methodological safeguards effectively mitigated potential confounding effects and substantially addressed sample overlap concerns inherent in UK Biobank-derived datasets.

### Statistical analysis

#### MR analysis

A valid MR study must satisfy three core assumptions: (1) a strong and robust association between IVs and the exposure; (2) independence of IVs from confounders affecting the exposure-outcome relationship; and (3) genetic variants influencing the outcome exclusively through the exposure, without alternative pathways^[37]^. We employed six MR approaches to validate causal relationships between TyG and MASLD: inverse variance weighted (IVW), MR-Egger, weighted median, weighted mode, simple mode, and generalized summary-data MR (GSMR). The IVW method is considered the most accurate estimator under valid instrumental assumptions and serves as our primary analytical framework^[38,39]^. GSMR, implemented via the R package developed by Zhu *et al*^[40,41]^, enhanced causal inference by integrating pleiotropy-robust pruning and heterogeneity adjustment.

#### Sensitivity analysis

Sensitivity analyses were conducted to assess violations of horizontal pleiotropy and heterogeneity in MR estimates. Horizontal pleiotropy was evaluated via MR-Egger regression, with an intercept test *P* < 0.05 indicating significant pleiotropic bias^[42]^. Non-significant pleiotropy (*P* ≥ 0.05) supported the validity of IVs. Heterogeneity was quantified using Cochran’s *Q*-test, where *P* < 0.05 denoted substantial between-SNP heterogeneity^[43]^. For analyses with significant heterogeneity (*Q*-test *P* < 0.05), random-effects IVW models were applied; otherwise, fixed-effects IVW models were employed^[44]^.

#### Comparative evaluation of four TWAS frameworks for TyG and MASLD

We integrated MAGMA due to its robust framework for gene and gene-set association testing. It aggregates SNP-level signals (*P* < 5 × 10^−8^) into gene-level associations using principal component regression, which explicitly models linkage disequilibrium (LD) and mitigates confounding by gene size^[25]^. This approach detects multi-marker effects often missed by SNP-wise methods. MAGMA’s two-tiered architecture – separating gene analysis from gene-set testing – ensures flexibility and statistical efficiency, enabling reliable identification of biological pathways^[25]^. We developed JTI-PrediXcan to integrate cross-tissue regulatory similarity (expression and epigenomic profiles) through elastic net optimization, training multi-tissue prediction models on GTEx v8 eQTL weights across 49 tissues. This framework enhances association power by borrowing information from biologically related tissues, particularly for underpowered tissues^[29,30]^. SMulTiXcan enhances cross-tissue gene detection by meta-analyzing tissue-specific Summary-PrediXcan (S-PrediXcan) results^[45]^. It leverages Multi-trait Adaptive Shrinkage (MASHR) effect models from GTEx v8 and corrects inter-tissue correlation via a SNP covariance matrix^[46]^. This boosts power to detect genes with distributed or synergistic effects across tissues, addresses single-tissue limitations, and reduces false positives^[29,45,46]^. FOCUS employs a Bayesian fine-mapping framework to probabilistically prioritize causal genes within GWAS risk loci. It addresses a key limitation of standard TWAS: LD induces spurious gene–trait associations at noncausal genes near causal variants^[32]^. By modeling the correlation structure of TWAS signals – driven by LD and eQTL weights – FOCUS computes posterior inclusion probabilities (PIPs) and generates ρ-credible gene sets^[32]^. These sets represent genes with a defined probability of containing the causal gene(s) explaining the association signal^[32,47,48]^. Consensus genes required agreement across two or more TWAS methods. When different methods – each with distinct underlying assumptions and approaches (MAGMA’s gene-level signal aggregation, JTI-PrediXcan/SMulTiXcan’s tissue-specific modeling, and FOCUS’s causal fine-mapping) – converge on the same gene–trait association, it strongly suggests that the finding is robust and less likely to be a technical artifact or false positive specific to any single method’s limitations. This concordance effectively mitigates the individual weaknesses of each approach and provides independent validation, collectively ensuring the identification of biologically plausible gene targets with higher confidence. *P*-values were adjusted using the false discovery rate (FDR) method to account for potential correlations among phenotypes, as the Bonferroni correction was deemed overly conservative^[49]^. The final set combines TyG- and MASLD-associated genes meeting this reproducibility threshold, highlighting loci linked to both traits. Detailed methods are in Supplemental Digital Content Table S1, available at: http://links.lww.com/JS9/F463.

#### Independent validation of comorbidity genes using FUSION TWAS framework for MASLD pathogenesis

To confirm the robustness of TyG-MASLD comorbidity genes identified through multi-method consensus, we performed independent validation using the FUSION TWAS platform on the MASLD GWAS dataset. The FUSION framework (Supplemental Digital Content Table S1, available at: http://links.lww.com/JS9/F463) was employed to systematically integrate GWAS summary statistics with tissue-specific eQTL data, enabling TWAS to identify genes mechanistically linked to MASLD^[26]^. This platform leverages multiple predictive models to estimate SNP-based weights for gene expression, thereby capturing polygenic effects while addressing tissue heterogeneity through cross-tissue meta-analyses^[26]^. For discovery-phase analyses, genome-wide significant SNPs (*P* < 5 × 10^−8^) were prioritized, followed by LD clumping (*r*^2^ < 0.001) to ensure independence of IVs^[26]^. Stringent thresholds were applied to control false discoveries, including FDR for multi-test correction. FUSION enables orthogonal validation by using summary data and reference panels, improving biological interpretability while reducing confounding.

#### Validation of TyG-MASLD comorbidity genes using PoPS framework

We validated TyG-MASLD comorbidity genes using PoPS, which integrates GWAS data with multi-omics to prioritize causal genes. PoPS (Supplemental Digital Content Table S1, available at: http://links.lww.com/JS9/F463) leverages polygenic enrichments across 57 543 gene features – including single-cell RNA-seq expression profiles, pathway annotations, and protein–protein interaction networks – to assign priority scores reflecting shared functional characteristics of causal genes^[33]^. Key strengths: tissue-specific modeling, reduced false positives via L2 regularization, and genome-wide prioritization^[33]^. We applied PoPS to TyG/MASLD data using genome-wide significant SNPs (*P* < 5 × 10^−8^) and ancestry-matched LD panels. Its cross-trait architecture integration – validated at 74% precision – identified shared pathogenic mechanisms^[33]^. Intersecting PoPS-prioritized genes with consensus loci validated targets with convergent transcriptomic evidence. This resolved the pleiotropy/tissue heterogeneity limitations of single-method approaches.

#### Single-cell spatially resolved transcriptomic characterization of TyG-MASLD shared genes in MASLD

To systematically investigate the co-pathogenic genes and tissue-specific spatial distribution patterns linking the TyG index and MASLD, this study employed the genetically informed spatial mapping (gsMap) method, published in Nature in 2025^[34]^. This approach integrates sc-ST data with GWAS statistics for MASLD. The gsMap (Supplemental Digital Content Table S1, available at: http://links.lww.com/JS9/F463) framework leverages a graph neural network (GNN) to harmonize gene expression profiles, spatial coordinates, and GWAS-derived trait associations^[34]^. It evaluates heritability enrichment using stratified linkage LD score regression (S-LDSC) and quantifies disease-region associations via the Cauchy combination test^[34]^. By overcoming the spatial resolution limitations of conventional single-cell RNA sequencing, gsMap enables precise mapping of spatially resolved cell populations associated with complex traits. Using mouse embryos (E16.5, 25 organs) and human GWAS data, we generated single-cell spatial maps of TyG-MASLD co-pathogenic genes. Cross-species validation provided spatial multiomics evidence for TyG-MASLD comorbidity mechanisms.

## Results

### MR analysis and sensitivity analysis results

MR analysis revealed a significant causal association between the TyG and MASLD (Supplemental Digital Content Table S3, available at: http://links.lww.com/JS9/F463). Using the IVW method, we observed a positive causal effect (OR = 1.58, 95% CI: 1.04–2.38, *P* = 0.030), supported by consistent findings from GSMR analysis (OR = 1.43, 95% CI: 1.27–1.61, *P* = 5.20 × 10^−9^). Sensitivity analyses identified significant heterogeneity across IVs (Cochran’s *Q P* = 1.88 × 10^−42^), prompting the use of a multiplicative random-effects IVW model, which retained significance (OR = 1.58, 95% CI: 1.04–2.38, *P* = 0.030). Horizontal pleiotropy was not detected (MR-Egger intercept *P* = 0.102), confirming the robustness of the causal estimates. These results underscore TyG as a genetically driven risk factor for MASLD, independent of confounding pathways.

### Comparative evaluation of four TWAS frameworks for TyG and MASLD

**Figure 2.**
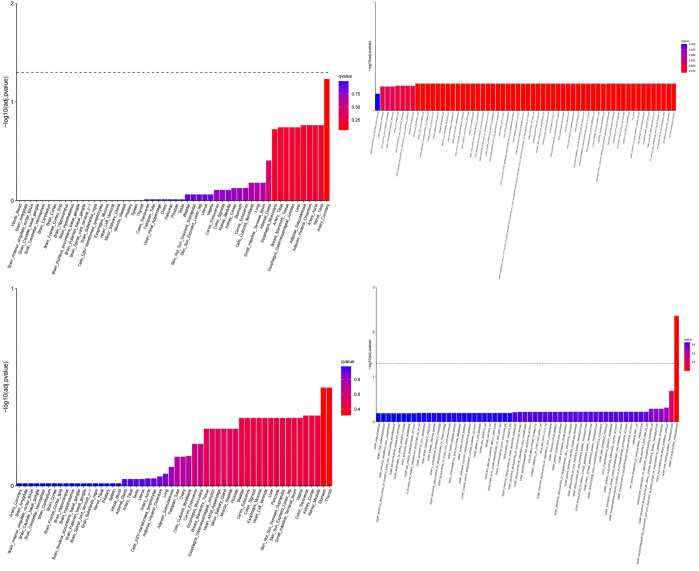
MAGMA-based tissue-specific enrichment and pathway enrichment analyses for TyG and MASLD. This composite figure presents MAGMA results comparing TyG (top row) and MASLD (bottom row). Left panels show tissue-specific enrichment: TyG was significantly enriched in adipose, vascular tissues, and liver, while MASLD showed enrichment in gastrointestinal and kidney tissues. Right panels show the top enriched biological pathways: TyG pathways centered on lipid metabolism and stress responses, whereas MASLD pathways are involved in developmental morphogenesis and immune signaling. These distinct enrichment patterns highlight the differing tissue involvements and underlying biological mechanisms between the TyG index and MASLD. TyG, triglyceride-glucose index; MASLD, metabolic dysfunction-associated steatotic liver disease.

**Figure 3.**
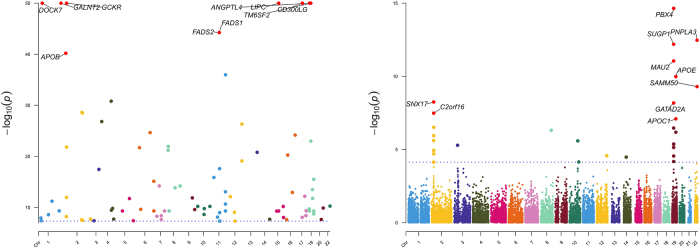
Manhattan Plots of MAGMA-Based Positive Gene Selection for TyG (Left) and MASLD (Right). Genes surpassing the FDR-adjusted significance threshold (*P* < 0.05) are highlighted. Key metabolic regulators TM6SF2 and GCKR exhibit genome-wide significance in both traits, linking insulin resistance (TyG) to hepatic lipid dysregulation (MASLD). Notably, PNPLA3 is uniquely prioritized in MASLD, underscoring its established role in steatosis progression. Vertical dashed lines indicate chromosome boundaries. TyG, triglyceride-glucose index; MASLD, metabolic dysfunction-associated steatotic liver disease.

**Figure 4.**
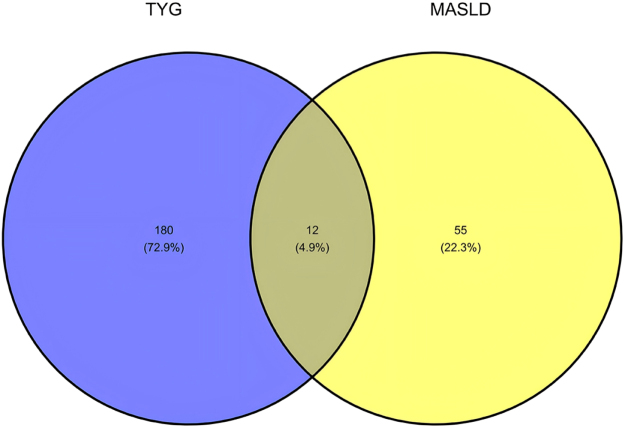
Shared candidate genes between TyG and MASLD identified by transcriptomic analysis. The Venn diagram illustrates the 12 comorbid genes consistently identified by at least two independent transcriptomic methodologies. Variation in the number of supporting methods per gene (detailed in Table 1) reflects differences in tissue-specific expression patterns and underlying genetic effect sizes. Genes validated by more frameworks (eg, TM6SF2, GCKR) typically exhibit stronger associations and broader tissue relevance, highlighting their heightened biological significance in the TyG-MASLD comorbidity pathway. TyG, triglyceride-glucose index; MASLD, metabolic dysfunction-associated steatotic liver disease.

We first analyzed the similarities and differences in the tissue-specific enrichment of TyG and MASLD using MAGMA (Supplemental Digital Content Table S4, available at: http://links.lww.com/JS9/F463 and Fig. 2). TyG exhibited significant enrichment (*P* < 0.05) in adipose-related tissues (Adipose Subcutaneous: *P* = 0.012; Adipose Visceral Omentum: *P* = 0.013), vascular tissues (Artery Aorta: *P* = 0.016; Artery Coronary: *P* = 0.0011; Artery Tibial: *P* = 0.030), and liver (*P* = 0.026). In contrast, MASLD showed enrichment in gastrointestinal tissues (Colon Transverse: *P* = 0.027; Stomach: *P* = 0.012; Small Intestine Terminal Ileum: *P* = 0.061) and kidney regions (Kidney Cortex: *P* = 0.041; Kidney Medulla: *P* = 0.036). Both traits shared no overlapping significant tissues. Notably, TyG demonstrated positive associations (BETA > 0) in enriched tissues (e.g., Artery Coronary: BETA = 1.508), whereas MASLD displayed mixed directions (e.g., Colon Transverse: BETA = 0.022; Artery Coronary: BETA = − 0.006). Neural and reproductive tissues showed no significance (*P* > 0.05) in either analysis. We then used MAGMA to compare TyG with MASLD pathway enrichment (see Supplemental Digital Content Tables S5 and S6, available at: http://links.lww.com/JS9/F463), after which we plotted the top 60 most significant enriched pathways (see Fig. 2 for the results). TyG demonstrated significant enrichment in pathways related to lipid catabolism, lipid homeostasis, and apoptosis regulation. Additionally, oxidative stress responses and PPAR signaling were prominent. In contrast, MASLD exhibited enrichment in pathways associated with developmental morphogenesis, immune regulation, and small Guanosine Triphosphatase (GTPase)-mediated signal transduction. Notably, MASLD highlighted neurotrophic signaling and calcium ion transport regulation. No overlapping pathways were observed between TyG and MASLD. TyG uniquely emphasized metabolic processes, whereas MASLD prioritized cellular communication and tissue-specific developmental pathways. Finally, we performed MAGMA-based positive gene selection for TyG and MASLD separately, selecting genes with FDR-adjusted *P*-values < 0.05 as the respective positive gene sets (Supplemental Digital Content Table S7, available at: http://links.lww.com/JS9/F463 and Fig. 3). These gene sets were subsequently incorporated into our comorbidity gene analysis pipeline. Subsequently, we conducted JTI-PrediXcan, SMulTiXcan, and FOCUS analyses for TyG and MASLD. The JTI-PrediXcan results for TyG and MASLD are detailed in Supplemental Digital Content Tables S8 and S9, available at: http://links.lww.com/JS9/F463, respectively; the SMulTiXcan results in Supplemental Digital Content Tables S10 and S11, available at: http://links.lww.com/JS9/F463; and the FOCUS analysis outcomes in Supplemental Digital Content Tables S12 and S13, available at: http://links.lww.com/JS9/F463. Notably, the JTI-PrediXcan analysis incorporates three models (JTI, Unified Test for Molecular Signatures (UTMOST), and PrediXcan) under a unified algorithm. In contrast, SMulTiXcan, while utilizing the same software as JTI-PrediXcan, employs a distinct methodology requiring GWAS data imputation and implementation of the Multivariate Adaptive Shrinkage (MASHR) prediction model. For FOCUS analysis, we applied a genome-wide significance threshold (5 × 10^−8^) at the SNP level and selected a stringent PIP threshold of 0.8 based on empirical criteria. Following the analysis with the four methodologies, we compiled comprehensive positive gene sets for both TyG and MASLD based on predefined significance thresholds, with methodological annotations provided in Supplemental Digital Content Tables S14 and S15, available at: http://links.lww.com/JS9/F463. To identify shared candidate genes between TyG and MASLD, we applied a stringent criterion requiring validation by at least two independent transcriptomic approaches. The resulting overlapping genes are summarized in Table 1 and illustrated in Figure 4. Our multi-method transcriptomic analysis identified 12 genes (C2orf16/SPATA31H1, FNDC4, GCKR, GMIP, HAPLN4, LPAR2, MAU2, MEF2B, NDUFA13, NRBP1, TM6SF2, and ZNF513) consistently supported by at least two approaches in both TyG and MASLD studies. Although all four methodologies were applied to both analyses, the methodological convergence differed between TyG and MASLD. In TyG, gene associations were predominantly validated by JTI-PrediXcan and SMulTiXcan, with no genes showing significance across more than two methods. In contrast, MASLD demonstrated enhanced methodological concordance, with several genes (e.g., TM6SF2, GMIP, and HAPLN4) supported by three or four approaches, including MAGMA and FOCUS, which contributed uniquely to MASLD-specific validations.

**Table 1:** List of shared candidate genes between TyG and MASLD identified by at least two of four transcriptomic methodologies

Gene	Methods (TyG)	Methods (MASLD)
C2orf16/SPATA31H1	JTI-PrediXcan, SMulTiXcan	MAGMA, JTI-PrediXcan, SMulTiXcan
FNDC4	JTI-PrediXcan, SMulTiXcan	JTI-PrediXcan, SMulTiXcan
GCKR	MAGMA, JTI-PrediXcan, SMulTiXcan	MAGMA, FOCUS, JTI-PrediXcan
GMIP	JTI-PrediXcan, SMulTiXcan	MAGMA, FOCUS, JTI-PrediXcan, SMulTiXcan
HAPLN4	JTI-PrediXcan, SMulTiXcan	MAGMA, FOCUS, JTI-PrediXcan, SMulTiXcan
LPAR2	JTI-PrediXcan, SMulTiXcan	FOCUS, JTI-PrediXcan, SMulTiXcan
MAU2	JTI-PrediXcan, SMulTiXcan	MAGMA, FOCUS, JTI-PrediXcan
MEF2B	JTI-PrediXcan, SMulTiXcan	MAGMA, SMulTiXcan
NDUFA13	JTI-PrediXcan, SMulTiXcan	FOCUS, JTI-PrediXcan
NRBP1	JTI-PrediXcan, SMulTiXcan	FOCUS, JTI-PrediXcan, SMulTiXcan
TM6SF2	MAGMA, SMulTiXcan	MAGMA, FOCUS, JTI-PrediXcan, SMulTiXcan
ZNF513	JTI-PrediXcan, SMulTiXcan	FOCUS, JTI-PrediXcan, SMulTiXcan

MAGMA, Multi-marker Analysis of GenoMic Annotation; JTI-PrediXcan, Joint-Tissue Imputation PrediXcan; SMulTiXcan, Sparse Multi-Tissue PrediXcan; FOCUS, Fine-mapping of Causal Gene Sets; TyG, triglyceride-glucose index; MASLD, metabolic dysfunction-associated steatotic liver disease.

### Independent validation of comorbidity genes using FUSION TWAS framework for MASLD pathogenesis

Using the FUSION method for independent validation of TyG-MASLD comorbid genes, nine genes (GMIP, HAPLN4, LPAR2, MAU2, MEF2B, NDUFA13, NRBP1, TM6SF2, and ZNF513) were successfully validated with significant *P*_fdr_ values (Supplemental Digital Content Table S16, available at: http://links.lww.com/JS9/F463). Specifically, GMIP showed associations in the Brain Amygdala (*P*_fdr_ = 0.0356) and Minor Salivary Gland (*P*_fdr_ = 0.0440). HAPLN4 exhibited significant signals in the Colon Sigmoid (*P*_fdr_ = 0.0344) and Minor Salivary Gland (*P*_fdr_ = 9.78 × 10^−9^). LPAR2 was validated in the Brain Amygdala (*P*_fdr_ = 8.22 × 10^−7^). MAU2 demonstrated significance in Adipose Subcutaneous tissue (*P*_fdr_ = 6.97 × 10^−5^). MEF2B was associated in the Brain Frontal Cortex BA9 (*P*_fdr_ = 2.60 × 10^−8^). NDUFA13 showed a significant signal in the Brain Caudate basal ganglia (*P*_fdr_ = 8.34 × 10^−7^). NRBP1 was validated in the Adrenal Gland (*P*_fdr_ = 0.0148). TM6SF2 exhibited significance in the Brain Caudate basal ganglia (*P*_fdr_ = 8.25 × 10^−7^), and ZNF513 was associated in the Brain Caudate basal ganglia (*P*_fdr_ = 0.0014).

The PoPS analysis of 12 comorbid genes associated with TyG and MASLD revealed both concordant and divergent directional associations, as reflected by the sign of the scores (Supplemental Digital Content Tables S17 and S18, available at: http://links.lww.com/JS9/F463, Table 2). In TyG, TM6SF2 (7.2491) and GCKR (6.7102) exhibited strongly positive scores, suggesting direct contributions to TyG regulation, whereas FNDC4 (0.7042), ZNF513 (0.3129), C2orf16/SPATA31H1 (0.2345), and NRBP1 (0.0609) displayed weaker positive associations. Conversely, GMIP (−0.7003), MEF2B (−0.7089), MAU2 (−0.4518), LPAR2 (−0.2991), NDUFA13 (−0.3693), and HAPLN4 (−0.003) showed negative scores in TyG, implying potential inhibitory roles or inverse relationships. In MASLD, the majority of genes – FNDC4 (−0.2012), LPAR2 (−0.2931), C2orf16/SPATA31H1(−0.2745), MAU2 (−0.2123), NDUFA13 (−0.5028), NRBP1 (−0.3126), ZNF513 (−0.1206), and HAPLN4 (−0.1853) – retained negative associations, with TM6SF2 (−0.0416) also shifting to a negative score. Notably, MEF2B (0.2001) and GMIP (0.0366) reversed their directional associations in MASLD, transitioning from negative in TyG to weakly positive, whereas GCKR (0.0588) maintained a marginally positive score. These sign discrepancies highlight both shared and opposing regulatory mechanisms across traits, with only GCKR preserving consistent positive directionality and NDUFA13 remaining negative in both.

**Table 2:** PoPS score analysis and comparison between TyG and MASLD

Gene	PoPS score (TyG)	PoPS score (MASLD)
C2orf16/SPATA31H1	0.2345	−0.2745
FNDC4	0.7042	−0.2012
GCKR	6.7102	0.0588
GMIP	−0.7003	0.0366
HAPLN4	−0.0030	−0.1853
LPAR2	−0.2991	−0.2931
MAU2	−0.4518	−0.2123
MEF2B	−0.7089	0.2001
NDUFA13	−0.3693	−0.5028
NRBP1	0.0609	−0.3126
TM6SF2	7.2491	−0.0416
ZNF513	0.3129	−0.1206

PoPS, Polygenic Priority Score; TyG, triglyceride-glucose index; MASLD, metabolic dysfunction-associated steatotic liver disease.

When evaluating the magnitude of associations (absolute PoPS scores), TyG exhibited markedly stronger polygenic signals compared to MASLD. In TyG, TM6SF2 (7.2491) and GCKR (6.7102) demonstrated exceptionally high absolute scores (>5), followed by FNDC4 (0.7042), GMIP (0.7003), MEF2B (0.7089), and NDUFA13 (0.3693), all exceeding 0.3. In contrast, MASLD-associated scores were uniformly low, with NDUFA13 (0.5028) and MEF2B (0.2001) ranking highest but still below 0.6, followed by LPAR2 (0.2931), C2orf16/SPATA31H1 (0.2745), MAU2 (0.2123), FNDC4 (0.2012), and HAPLN4 (0.1853), whereas TM6SF2 (0.0416) and GCKR (0.0588) showed minimal magnitudes.

Ranking prioritization based on absolute scores further emphasized cross-trait differences. For TyG, the order was: (1) TM6SF2 (7.2491), (2) GCKR (6.7102), (3) MEF2B (0.7089), (4) FNDC4 (0.7042), (5) GMIP (0.7003), (6) NDUFA13 (0.3693), (7) MAU2 (0.4518), (8) ZNF513 (0.3129), (9) LPAR2 (0.2991), (10) C2orf16/SPATA31H1 (0.2345), (11) NRBP1 (0.0609), and (12) HAPLN4 (0.003). In MASLD, the hierarchy was: (1) NDUFA13 (0.5028), (2) LPAR2 (0.2931), (3) C2orf16/SPATA31H1 (0.2745), (4) NRBP1 (0.3126), (5) MAU2 (0.2123), (6) FNDC4 (0.2012), (7) MEF2B (0.2001), (8) HAPLN4 (0.1853), (9) ZNF513 (0.1206), (10) GCKR (0.0588), (11) TM6SF2 (0.0416), and (12) GMIP (0.0366). Key divergences included TM6SF2 and GCKR, which dominated TyG rankings (1st and 2nd) but fell to the lowest positions in MASLD (11th and 10th), and NDUFA13, which rose from 6th in TyG to 1st in MASLD. MEF2B maintained moderate priority (3rd in both), whereas FNDC4 and MAU2 showed comparable mid-tier ranks (4th/6th and 7th/5th, respectively).

### Single-cell spatially resolved transcriptomic characterization of TyG-MASLD shared genes in MASLD

**Figure 5.**
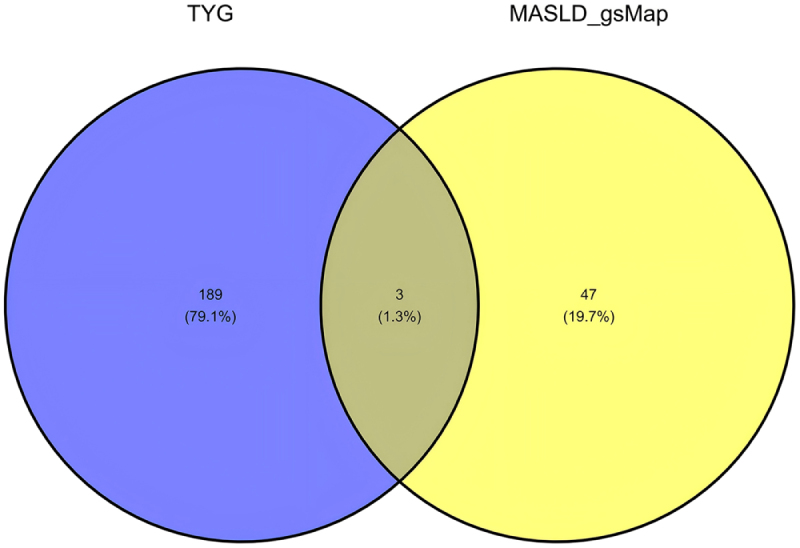
Cross-species validation of TyG-MASLD comorbidity genes via spatially resolved transcriptomics. The Venn diagram identifies evolutionarily conserved genes (APOA1, APOB, and APOC4) detected by gsMap analysis in embryonic mouse tissues (E16.5), despite the absence of the original 12 human comorbid genes. These apolipoproteins – critical regulators of lipid metabolism – emerged as spatially enriched hubs in liver/vascular microenvironments, highlighting their conserved role in hepatic lipid trafficking and systemic metabolic dysregulation. Their validation underscores species-invariant pathways as high-priority therapeutic targets. GsMap, genetically informed spatial mapping; TyG, triglyceride-glucose index; MASLD, metabolic dysfunction-associated steatotic liver disease.

**Figure 6.**
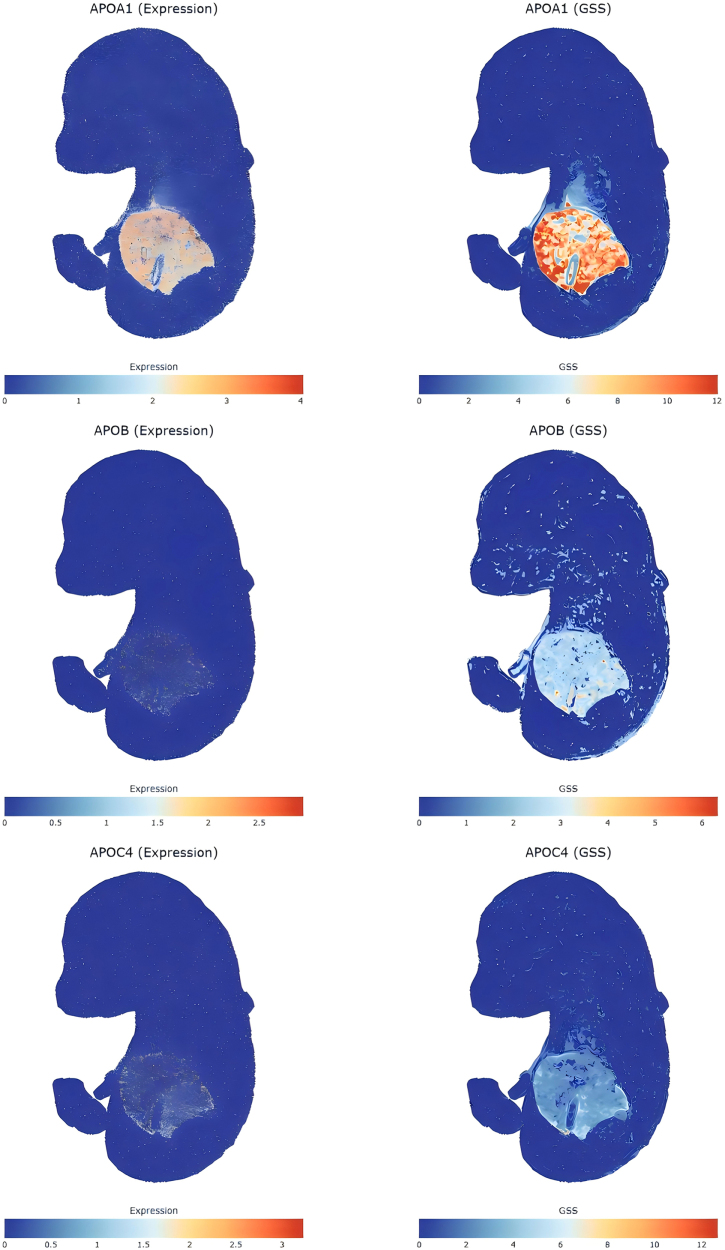
Spatial expression and GSS of MASLD-associated and TyG comorbid genes in mouse embryos mapped via gsMap (Supplemental Digital Content Materials, available at: http://links.lww.com/JS9/F420). GsMap, genetically informed spatial mapping; TyG, triglyceride-glucose index; MASLD, metabolic dysfunction-associated steatotic liver disease; GSS, Gene-Specific Scores. This figure illustrates the spatial expression profiles and GSS distributions of genes associated with MASLD and comorbid with TyG in embryonic mouse tissues. The analysis, performed using the gsMap method, highlights regions of high genetic activity (spots) where these genes exhibit significant expression. GSS values reflect localized gene expression rankings, emphasizing spatial heterogeneity in metabolic pathways relevant to MASLD and TyG comorbidity.

Our integrative analysis of sc-ST and GWAS data for MASLD in embryonic mice (E16.5, spanning 25 organs) revealed critical insights. First, none of the 12 comorbid genes previously validated for TyG and MASLD in humans showed significant expression across >120 000 cells in the mouse organs (Supplemental Digital Content Table S22, available at: http://links.lww.com/JS9/F463). This discrepancy may reflect species-specific differences in gene regulation or metabolic pathway divergence between humans and mice. However, we identified three additional genes – APOA1, APOB, and APOC4 – that exhibited significant expression in both TyG-associated gene sets (derived from multiple methods) and the mouse MASLD-positive gene set (see Supplemental Digital Content Table S22, available at: http://links.lww.com/JS9/F463, Figs. 5 and 6).

**Figure 7.**
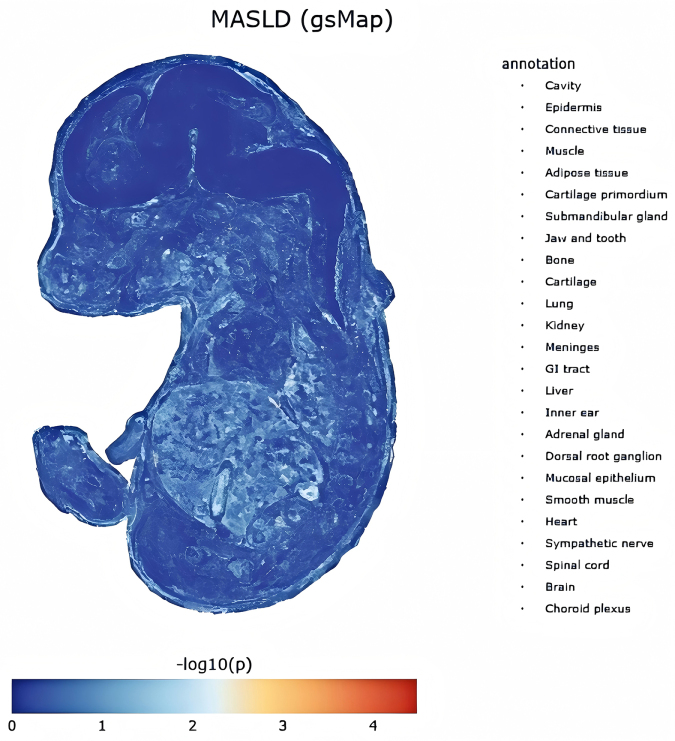
‘Spatial mapping of MASLD-associated cellular patterns in E16.5 mouse embryonic single-cell spatial transcriptomics data, generated by the gsMap algorithm across 25 organs (Supplemental Digital Content Materials, available at: http://links.lww.com/JS9/F419). GsMap of cells for complex traits. GsMap, genetically informed spatial mapping; MASLD, metabolic dysfunction-associated steatotic liver disease.

Next, spatial resolution analysis using sc-ST (Supplemental Digital Content Tables S19–S21, available at: http://links.lww.com/JS9/F463, Fig. 7) identified organs with localized MASLD-associated signals. The liver showed the highest density of positive spatial coordinates (*P* = 6.43 × 10^−5^), followed by connective tissue (*P* = 3.29 × 10^−5^), adrenal gland (*P* = 0.0004), lung (*P* = 0.0001), kidney (*P* = 0.0006), mucosal epithelium (*P* = 0.0023), GI tract (*P* = 0.0006), bone (*P* = 0.0024), cartilage primordium (*P* = 0.0037), jaw and tooth (*P* = 0.0029), and cavity (*P* = 0.0001). The liver’s dominance in spatial enrichment and cellular density underscores its central role in MASLD pathology.

In contrast, Cauchy testing (Table 3) identified only the adrenal gland as statistically significant (Cauchy *P* = 0.0064). Other spatially enriched organs, including the liver (Cauchy *P* = 0.0797), lung (Cauchy *P* = 0.0833), connective tissue (Cauchy *P* = 0.0745), and GI tract (Cauchy *P* = 0.0532), failed to reach significance. This divergence highlights methodological differences: sc-ST detects localized cellular-level signals, whereas Cauchy testing evaluates organ-wide associations, which may dilute spatially concentrated effects. For example, the liver’s high spatial signal density did not achieve organ-level significance, likely due to regional heterogeneity or insufficient statistical power in bulk-tissue analysis. The adrenal gland’s unique significance in both analyses aligns with its systemic hormonal role in metabolic regulation, a key feature of MASLD pathophysiology.

**Table 3:** Statistical validation of tissue-specific MASLD enrichment across spatially resolved murine organ regions using the gsMap algorithm with Cauchy combination tests

Annotation	*P*_cauchy_	*P*_median_
Adrenal gland	0.0064	0.0219
GI tract	0.0532	0.1472
Connective tissue	0.0745	0.3421
Liver	0.0797	0.1646
Lung	0.0833	0.2163
Epidermis	0.0995	0.2300
Submandibular gland	0.1275	0.2783
Adipose tissue	0.1488	0.3808
Kidney	0.1987	0.3033
Mucosal epithelium	0.3043	0.4404
Muscle	0.3116	0.4722
Smooth muscle	0.3224	0.3842
Cartilage	0.3520	0.4277
Meninges	0.3547	0.4464
Inner ear	0.4531	0.4956
Choroid plexus	0.5987	0.5566
Bone	0.6094	0.5017
Jaw and tooth	0.6173	0.5499
Cartilage primordium	0.6662	0.5643
Sympathetic nerve	0.6828	0.5739
Dorsal root ganglion	0.7150	0.6323
Heart	0.9642	0.6286
Brain	0.9727	0.9237
Cavity	0.9778	0.4556
Spinal cord	0.9900	0.9315

MASLD, metabolic dysfunction-associated steatotic liver disease. The annotation denotes 25 embryonic mouse organs, *P*_cauchy_ represents the *P*-value from the Cauchy combination test, and *P*_median_ indicates the *P*-value derived from the median-based validation test.

Organs such as cartilage primordium (sc-ST *P* = 0.0037; Cauchy *P* = 0.6662) and cavity (sc-ST *P* = 0.0001; Cauchy *P* = 0.9778) further exemplified this contrast, where localized signals lacked organ-wide statistical support. Conversely, epidermis (Cauchy *P* = 0.0995) and submandibular gland (Cauchy *P* = 0.1275), though not prominent in sc-ST mapping, showed moderate but nonsignificant Cauchy *P*-values. These findings emphasize the complementary utility of sc-ST (for spatial hotspots) and Cauchy testing (for systemic associations).

## Discussion

Integrative genomics identified causal links and shared mechanisms between the TyG index and MASLD. MR using 192 SNPs established causality: genetically predicted TyG increased MASLD risk. Multi-method transcriptomics (MAGMA, JTI-PrediXcan, SMulTiXcan, and FOCUS) identified 12 consensus comorbidity genes (e.g., TM6SF2 and GCKR), validated independently (FUSION and PoPS) despite trait directional heterogeneity. Embryonic mouse sc-ST analysis revealed that while these 12 human genes lacked significant expression, three evolutionarily conserved lipid metabolism genes (APOA1, APOB, and APOC4) emerged as cross-species candidates, highlighting conserved metabolic dysregulation pathways.

Our study bridges a critical gap left by prior epidemiological work focused on observational TyG-MASLD associations^[50]^. Our genetic approach confirms TyG’s multiorgan relevance – supported by clinical evidence of vascular atherosclerosis^[51]^ and gut-liver axis involvement^[52]^ – while revealing key distinctions. Unlike hypertension studies linking TyG to kidney disease^[53]^, our MR demonstrates that renal signals are secondary to systemic inflammation/renin-angiotensin-aldosterone system (RAAS) activation. Further diverging from cumulative biomarker innovations^[54]^, our eQTL network identifies APOB-modulated lipid trafficking as an upstream genetic trigger. Our spatial mapping reveals adrenal glucocorticoid-catecholamine dysregulation within pro-fibrotic connective tissue microenvironments – a finding unattainable via circulating biomarkers^[55]^. Methodological distinctions explain discrepancies: TWAS captures dynamic gene–environment interactions (e.g., GCKR-diet), single-cell resolution reveals occult crosstalk, and genetic instruments mitigate confounders. This transcends correlative paradigms^[56]^, establishing TyG as a causal driver of MASLD-CVD comorbidity, revealing metabolic vulnerability priming and enabling genotype-guided therapy. TyG index is a clinically actionable, low-cost screening tool for early MASLD detection, especially where conventional models fail. Identified causal genes and apolipoprotein pathways offer theranostic targets; genotyping refines CVD risk stratification and prioritizes patients for targeted therapies. Key challenges include validating tissue-specific therapeutic windows, integrating polygenic risk scores, and developing TyG-genetic screening. Clinicians should implement longitudinal TyG monitoring, and genotype-stratified trials are needed. Integrating TyG-associated genetic variants with established noninvasive fibrosis biomarkers refines MASLD risk stratification by identifying individuals with heightened genetic susceptibility. This integrated approach enhances conventional screening tools, enabling earlier confirmatory imaging and prioritization for targeted therapies, optimizing precision prevention in high-risk cohorts.

The TyG, a surrogate marker of insulin resistance, contributes to the development and progression of MASLD via multiple mechanisms. Insulin resistance increases hepatic *de novo* lipogenesis while suppressing fatty acid β-oxidation, leading to abnormal lipid accumulation in hepatocytes^[16]^. Elevated TyG levels are also associated with systemic inflammation and enhanced oxidative stress, exacerbating hepatocellular injury and fibrosis^[18]^. Furthermore, TyG elevation directly promotes atherosclerosis via endothelial dysfunction and lipotoxicity, indirectly amplifying cardiovascular risks in MASLD patients^[8]^. Genetic studies demonstrate that TyG-associated genes influence hepatic lipid deposition by regulating lipid metabolism and gluconeogenic pathways^[27]^. Clinical evidence indicates that TyG positively correlates with MASLD severity and serves as a biomarker for predicting fibrosis progression^[20]^. In animal models, TyG elevation promotes hepatic steatosis by activating PPARγ and Sterol Regulatory Element-Binding Protein 1c (SREBP-1c) pathways^[19]^. Collectively, the causal relationship between TyG and MASLD involves multifactorial interactions encompassing insulin resistance, lipotoxicity, inflammation, and genetic regulation.

C2orf16/SPATA31H1’s function remains unknown. GWAS suggests it regulates lipid metabolism via nearby elements, influencing TyG and liver fat. PoPS reveals opposing effects: positive with TyG but negative with MASLD. This paradox likely arises as the gene desert-located C2orf16 acts tissue-specifically, impacting metabolic pathways differently in insulin-sensitive vs. steatotic tissues^[57]^. FNDC4, a fibronectin domain protein, likely regulates insulin sensitivity via fat browning or inflammation, with levels linked to liver fat^[58]^. Its positive TyG PoPS score (0.7042) aligns with adipocyte roles, whereas the negative MASLD score (−0.2012) suggests disease downregulation, possibly mitigating inflammatory damage^[59]^. This shift mirrors known changes in fat cell signaling during NAFLD^[59]^. GCKR, encoding glucokinase regulatory protein, regulates hepatic GLU metabolism and TG synthesis, and its rs1260326 polymorphism is strongly associated with elevated TyG index and increased MASLD risk^[60]^. Critically, GCKR has a strong positive PoPS score in TyG (6.7102) and a weak positive score in MASLD (0.0588), indicating a consistent effect promoting metabolic dysregulation. This reflects GCKR’s central role in liver GLU sensing and fat production, driving both hyperglycemia/hypertriglyceridemia (raising TyG) and liver fat buildup in MASLD. GMIP, a Rho GTPase regulator, may disrupt lipid homeostasis by affecting hepatocyte polarity and lipid droplet transport^[61]^. PoPS reveals a significant direction reversal for GMIP: negative in TyG (−0.7003) but positive in MASLD (0.0366), suggesting opposing functions in different disease stages. In early insulin resistance, reduced GMIP activity may impair cellular structure and fat movement, promoting harmful fat buildup. Conversely, in established MASLD, increased GMIP activity might represent an adaptive response, potentially restoring liver cell function or enabling fat storage, similar to cellular adaptations seen under fat stress^[59]^. HAPLN4 was identified as a key locus influencing hepatic steatosis in a large-scale multi-ethnic meta-analysis^[62]^. However, its comorbid mechanism linking the TyG index and MASLD remains uncharacterized, requiring experimental validation. Lysophosphatidic acid receptor 2 (LPAR2) mediates lysophosphatidic acid (LPA) signaling, promoting liver cell damage and inflammation. LPA signaling [particularly via lysophosphatidic acid receptor 1 (LPAR1)] drives fibrosis, and LPAR1 antagonists demonstrate anti-fibrotic potential^[63]^. LPA actions also promote many features of liver cancer^[64]^. Crucially, LPAR2 has negative PoPS scores in both TyG and MASLD, suggesting lower LPAR2 activity or expression may be protective. This aligns with LPAR2’s established pro-fibrotic/pro-inflammatory roles in liver disease. Suppressing its signaling could mitigate LPA-mediated damage, inflammation, and fibrosis throughout the TyG-MASLD spectrum^[65]^. MAU2, a component of the chromosomal condensin complex, has been associated with hepatic steatosis in normal-weight individuals through its genetic variants^[66]^. Studies have demonstrated that gene–gene and gene–environment interactions within the NCAN-TM6SF2-CILP2-PBX4-SUGP1-MAU2 locus significantly influence hyperlipidemia^[67]^. MAU2 has negative PoPS scores in both TyG (−0.4518) and MASLD (−0.2123), suggesting reduced function may promote lipid problems and liver fat. This may occur via indirect effects on chromatin organization and gene expression at its locus, impacting lipid-regulating genes. NDUFA13, a nuclear-encoded subunit of mitochondrial complex I (NADH: ubiquinone oxidoreductase), maintains essential oxidative phosphorylation (OXPHOS) function. When NDUFA13 is impaired due to genetic mutations, posttranslational modifications, or oxidative damage, it induces mitochondrial dysfunction. In hepatocytes, this triggers a vicious cycle: energy deficiency and oxidative stress disrupt insulin signaling, exacerbating hepatic insulin resistance. These pathological changes contribute to the development of metabolic disorders^[68,69]^. The TM6SF2 rs58542926 mutation impairs liver with very-low-density lipoprotein (VLDL) secretion^[70]^. This causes fat buildup in liver cells (steatosis) and abnormal blood lipids, promoting fatty liver disease and heart risks^[70]^. PoPS analysis shows a striking contrast: TM6SF2 has a strong positive score in TyG (7.2491) but a weak negative score in MASLD (−0.0416). This highlights tissue-specific effects. In TyG, the mutation directly raises blood TGs (positive score) by blocking VLDL secretion, driving the TyG index. However, in the liver, this mutation primarily causes severe steatosis. Its direct association with advanced MASLD complications appears attenuated, potentially modified by other factors, resulting in a weak negative correlation^[71]^. MEF2B shows opposing associations (TyG: −0.7089; MASLD: +0.2001). Reduced activity in peripheral tissues during insulin resistance impairs metabolic adaptation^[72]^, whereas elevated activity in steatosis drives pro-fibrogenic programs in hepatic stellate cells (HSCs) – consistent with its role in NASH-related macrophage chemotaxis^[73]^. NRBP1 shifts from weak positive (TyG: +0.0609) to negative (MASLD: −0.3126), suggesting dual functionality: mild support for growth factor signaling in early dysmetabolism^[74]^ *versus* failure of ubiquitin-dependent constraint on hepatic inflammation^[75]^. ZNF513 shows discordant weak effects (TyG: +0.3129; MASLD: −0.1206), suggesting context-dependent transcriptional regulation. It may modulate lipid metabolism genes in early disease but dysregulate stress-response pathways in chronic steatosis^[76,77]^.

The apolipoproteins APOA1, APOB, and APOC4 are mechanistically central to the comorbidity between the TyG index and MASLD. APOA1, APOB, and APOC4 are core members of the apolipoprotein family that play crucial roles in regulating lipid metabolism and insulin resistance. APOA1 serves as the primary structural protein of high-density lipoprotein^[78,79]^. Reduced APOA1 expression impairs reverse cholesterol transport, directly promoting hepatic lipid accumulation and exacerbating insulin resistance^[78,79]^ – core pathways in MASLD. APOB is the key structural component of atherogenic lipoproteins (low-density lipoprotein and VLDL). Its overexpression drives abnormal VLDL secretion from hepatocytes, elevating circulating TGs^[80–82]^, which worsens hepatic steatosis and insulin resistance^[81,82]^. APOC4, a member of the apolipoprotein C family, contributes to dysregulation by impairing plasma TG clearance efficiency, further promoting intrahepatic lipid deposition^[83–85]^. These genes maintain nonredundant roles in lipid homeostasis. Deleterious mutations cause severe dyslipidemias, driving strong purifying selection against nonfunctional variants. APOA1 has conserved domains essential for lipid binding and lecithin-cholesterol acyltransferase activation^[78]^. APOB contains indispensable regions for lipoprotein assembly under intense selective constraint^[80]^. APOC4 is in a conserved, tightly regulated apolipoprotein gene cluster. Thus, our sc-ST analysis in embryonic mouse tissue identified APOA1, APOB, and APOC4 as conserved nodes, though not all 12 human comorbidity genes. The other genes likely missed due to species differences in regulation, polygenic traits, or embryonic model limitations for human comorbidities^[86]^. Genes affecting risk through subtle regulation or gene–environment interactions often diverge more evolutionarily^[86]^. The conserved dysfunction in these apolipoproteins confirms the value of mouse models for core TyG-MASLD pathways. Key mechanisms – impaired fatty acid β-oxidation and faulty VLDL secretion driven by APOA1/APOB/APOC4, are recapitulated. Thus, despite species differences in some susceptibility genes, mouse models reliably reveal central lipid transport defects in TyG-linked MASLD, especially within these conserved pathways.

Our multi-omics framework identified tissue-specific signatures for the TyG index and MASLD. TyG’s strong adipose association reflects its function as an insulin resistance marker (based on TGs/GLU), influenced by adipose processes including lipolysis, adipokine secretion, and inflammation^[17]^. Vascular enrichment confirms the link between TyG, endothelial dysfunction, and atherosclerosis. Clinical studies show that TyG predicts cardiovascular events and arterial stiffness^[87]^. Gastrointestinal enrichment in MASLD highlights the critical gut-liver axis. Dysbiosis, increased gut permeability, and bacterial translocation drive hepatic inflammation and steatosis progression, as shown by clinical and translational research^[88,89]^. MASLD links to kidney disease, indicating shared metabolic risks and chronic kidney disease connections via systemic inflammation, insulin resistance, and RAAS activation. Embryonic sc-ST mapping localized signals to liver, connective tissue, and adrenal gland. The organ-wide Cauchy test was significant only in the adrenal gland, but liver/connective tissue spatial hotspots align with core MASLD pathology: hepatocyte lipid accumulation and HSC activation driving fibrosis^[4]^. The adrenal gland’s significance in both sc-ST density and Cauchy tests suggests an underappreciated endocrine role. Dysregulated glucocorticoid/catecholamine stress responses may drive metabolic-inflammatory changes, requiring clinical studies on adrenal-MASLD severity links.

Our study has limitations requiring consideration. The MR analysis was restricted to European-ancestry populations, limiting generalizability. TWAS utilized GTEx v8 postmortem expression data, potentially introducing tissue-degradation biases unrelated to *in vivo* metabolic states. TyG data were filtered to 192 pre-selected SNPs rather than raw measurements, which may yield incomplete TWAS results – though this constraint originates from the source data. Although identified genes represent therapeutic targets, their mechanistic roles in endothelial dysfunction require validation through single-cell sequencing, animal models, and multi-ethnic replication to guide clinical translation. Collectively, these limitations underscore the need for broader population sampling and functional genomics to advance precision management of TyG-MASLD.

## Conclusion

This study establishes the TyG index as a causal driver of MASLD. We identified 12 comorbid genes (e.g., TM6SF2 and GCKR) and conserved apolipoproteins (APOA1, APOB, and APOC4) that link hepatic lipid dysregulation to systemic cardiometabolic disturbances by orchestrating lipid trafficking, insulin signaling, and hepatic stress responses. These molecular hubs connect steatosis progression to cardiovascular risks, providing key insights into the unified etiology of CLMH disorders. Validating TyG as a causal biomarker and pinpointing these conserved pathways advances the CLMH field and highlights them as therapeutic targets. Clinically, integrating TyG-associated genetic variants with established noninvasive fibrosis biomarkers could enhance early identification of high-risk MASLD patients requiring intensified monitoring or confirmatory imaging. Future research should prioritize functional validation of these targets and explore combinatorial interventions targeting the TyG-MASLD-CVD axis to mitigate dual hepatic-cardiovascular risks.

## Data Availability

The R packages and Python-based software used in this study, along with their links and descriptions, are detailed in Supplemental Digital Content Table S1, available at: http://links.lww.com/JS9/F463. All SMR results and GWAS/QTL associations for the selected SNPs are provided in Supplemental Digital Content Tables S1–S22, available at: http://links.lww.com/JS9/F463. The original dataset and its links are provided in the content of the manuscript. We encourage interested readers to obtain data from their respective sources.

## References

[1] Stefan N Yki-Jarvinen H Neuschwander-Tetri BA. Metabolic dysfunction-associated steatotic liver disease: heterogeneous pathomechanisms and effectiveness of metabolism-based treatment. Lancet Diabetes Endocrinol 2025;13:134–48. 39681121 10.1016/S2213-8587(24)00318-8

[2] Ke Y Xu C Lin J Li Y. Role of hepatokines in non-alcoholic fatty liver disease. J Transl Int Med 2019;7:143–48. 32010600 10.2478/jtim-2019-0029 PMC6985917

[3] Colica C Abenavoli L. Resistin levels in non-alcoholic fatty liver disease pathogenesis. J Transl Int Med 2018;6:52–53. 29607306 10.2478/jtim-2018-0011 PMC5874489

[4] Rinella ME Lazarus JV Ratziu V. A multisociety delphi consensus statement on new fatty liver disease nomenclature. Ann Hepatol 2024;29:101133. 37364816 10.1016/j.aohep.2023.101133

[5] Brusnic O Onisor D Boicean A. Fecal microbiota transplantation: insights into colon carcinogenesis and immune regulation. J Clin Med 2024;13:6578. 39518717 10.3390/jcm13216578 PMC11547077

[6] Zanchetta M Adani GL Micheletti G. Perforated calculous cholecystitis and incidental squamous cell carcinoma of the gallbladder—a complex relationship with a difficult management in the acute setting. Medicina 2025;61:452. 40142263 10.3390/medicina61030452 PMC11944027

[7] Calomino N Carbone L Kelmendi E. Western experience of hepatolithiasis: clinical insights from a case series in a tertiary center. Medicina (Kaunas) 2025;61:860 40428818 10.3390/medicina61050860 PMC12113244

[8] Duell PB Welty FK Miller M. Nonalcoholic fatty liver disease and cardiovascular risk: a scientific statement from the american heart association. Arterioscler Thromb Vasc Biol 2022;42:e168–e85. 35418240 10.1161/ATV.0000000000000153

[9] Mantovani A Csermely A Petracca G. Non-alcoholic fatty liver disease and risk of fatal and non-fatal cardiovascular events: an updated systematic review and meta-analysis. Lancet Gastroenterol Hepatol 2021;6:903–13. 34555346 10.1016/S2468-1253(21)00308-3

[10] Lincoff AM Brown-Frandsen K Colhoun HM. Semaglutide and cardiovascular outcomes in obesity without diabetes. N Engl J Med 2023;389:2221–32. 37952131 10.1056/NEJMoa2307563

[11] Loomba R Hartman ML Lawitz EJ. Tirzepatide for metabolic dysfunction-associated steatohepatitis with liver fibrosis. N Engl J Med 2024;391:299–310. 38856224 10.1056/NEJMoa2401943

[12] Harrison SA Bedossa P Guy CD. A phase 3, randomized, controlled trial of resmetirom in NASH with liver fibrosis. N Engl J Med 2024;390:497–509. 38324483 10.1056/NEJMoa2309000

[13] McPherson S Hardy T Dufour JF. Age as a confounding factor for the accurate non-invasive diagnosis of advanced NAFLD fibrosis. Am J Gastroenterol 2017;112:740–51. 27725647 10.1038/ajg.2016.453 PMC5418560

[14] Chew NWS Pan XH Chong B Chandramouli C Muthiah M Lam CSP. Type 2 diabetes mellitus and cardiometabolic outcomes in metabolic dysfunction-associated steatotic liver disease population. Diabet Res Clin Pract 2024;211:111652. 10.1016/j.diabres.2024.111652 38574897

[15] Long MT Noureddin M Lim JK. AGA clinical practice update: diagnosis and management of nonalcoholic fatty liver disease in lean individuals: expert review. Gastroenterology 2022;163:764–74e1. 35842345 10.1053/j.gastro.2022.06.023 PMC9398982

[16] Guerrero-Romero F Simental-Mendia LE Gonzalez-Ortiz M. The product of triglycerides and glucose, a simple measure of insulin sensitivity. Comparison with the euglycemic-hyperinsulinemic clamp. J Clin Endocrinol Metab 2010;95:3347–51. 20484475 10.1210/jc.2010-0288

[17] Simental-Mendia LE Rodriguez-Moran M Guerrero-Romero F. The product of fasting glucose and triglycerides as surrogate for identifying insulin resistance in apparently healthy subjects. Metab Syndr Relat Disord 2008;6:299–304. 19067533 10.1089/met.2008.0034

[18] Luo E Wang D Yan G. High triglyceride-glucose index is associated with poor prognosis in patients with acute ST-elevation myocardial infarction after percutaneous coronary intervention. Cardiovasc Diabetol 2019;18:150. 31722708 10.1186/s12933-019-0957-3 PMC6852896

[19] Zhao S Yu S Chi C. Association between macro- and microvascular damage and the triglyceride glucose index in community-dwelling elderly individuals: the Northern Shanghai study. Cardiovasc Diabetol 2019;18:95. 31345238 10.1186/s12933-019-0898-x PMC6657056

[20] Rong L Hou N Hu J. The role of TyG index in predicting the incidence of diabetes in Chinese elderly men: a 20-year retrospective study. Front Endocrinol (Lausanne) 2023;14:1191090. 37424876 10.3389/fendo.2023.1191090 PMC10327477

[21] Yun Z Guo Z Li X. Genetically predicted 486 blood metabolites in relation to risk of colorectal cancer: a Mendelian randomization study. Cancer Med 2023;12:13784–99. 37132247 10.1002/cam4.6022 PMC10315807

[22] Zuccolo L Holmes MV. Commentary: mendelian randomization-inspired causal inference in the absence of genetic data. Int J Epidemiol 2017;46:962–65. 28025256 10.1093/ije/dyw327

[23] Richmond RC Davey Smith G. Mendelian randomization: concepts and scope. Cold Spring Harb Perspect Med 2022;12:a040501. 34426474 10.1101/cshperspect.a040501 PMC8725623

[24] Gamazon ER Wheeler HE Shah KP. A gene-based association method for mapping traits using reference transcriptome data. Nat Genet 2015;47:1091–98. 26258848 10.1038/ng.3367 PMC4552594

[25] de Leeuw CA Mooij JM Heskes T Posthuma D. MAGMA: generalized gene-set analysis of GWAS data. PLoS Comput Biol 2015;11:e1004219. 25885710 10.1371/journal.pcbi.1004219 PMC4401657

[26] Gui J Meng L Huang D. Identification of novel proteins for sleep apnea by integrating genome-wide association data and human brain proteomes. Sleep Med 2024;114:92–99. 38160582 10.1016/j.sleep.2023.12.026

[27] Si S Li J Li Y. Causal effect of the triglyceride-glucose index and the joint exposure of higher glucose and triglyceride with extensive cardio-cerebrovascular metabolic outcomes in the uk biobank: a mendelian randomization study. Front Cardiovasc Med 2020;7:583473. 33553250 10.3389/fcvm.2020.583473 PMC7863795

[28] Agha RA Mathew G Rashid R. Transparency In The reporting of Artificial INtelligence – the TITAN guideline. Premier Journal of Science 2025;10:100082.

[29] Liang Y Nyasimi F, and Im HK. Pervasive polygenicity of complex traits inflates false positive rates in transcriptome-wide association studies. bioRxiv [Preprint] 2024. doi:10.1101/2023.10.17.562831

[30] Zhou D Jiang Y Zhong X Cox NJ Liu C Gamazon ER. A unified framework for joint-tissue transcriptome-wide association and mendelian randomization analysis. Nat Genet 2020;52:1239–46. 33020666 10.1038/s41588-020-0706-2 PMC7606598

[31] Nikpay M Goel A Won HH. A comprehensive 1,000 genomes-based genome-wide association meta-analysis of coronary artery disease. Nat Genet 2015;47:1121–30. 26343387 10.1038/ng.3396 PMC4589895

[32] Mancuso N Freund MK Johnson R. Probabilistic fine-mapping of transcriptome-wide association studies. Nat Genet 2019;51:675–82. 30926970 10.1038/s41588-019-0367-1 PMC6619422

[33] Weeks EM Ulirsch JC Cheng NY. Leveraging polygenic enrichments of gene features to predict genes underlying complex traits and diseases. Nat Genet 2023;55:1267–76. 37443254 10.1038/s41588-023-01443-6 PMC10836580

[34] Song L Chen W Hou J Guo M Yang J. Spatially resolved mapping of cells associated with human complex traits. Nature 2025;641:932–41. 40108460 10.1038/s41586-025-08757-x PMC12095064

[35] Agha RA Mathew G Rashid R. Revised strengthening the reporting of cohort, cross-sectional and case-control studies in surgery (STROCSS) guideline: an update for the age of Artificial Intelligence. Premier Journal of Science 2025;10:100081.

[36] Chen R Petrazzini BO Duffy A. Trans-ancestral rare variant association study with machine learning-based phenotyping for metabolic dysfunction-associated steatotic liver disease. Genome Biol 2025;26:50. 40065360 10.1186/s13059-025-03518-5 PMC11892324

[37] Boef AG Dekkers OM le Cessie S. Mendelian randomization studies: a review of the approaches used and the quality of reporting. Int J Epidemiol 2015;44:496–511. 25953784 10.1093/ije/dyv071

[38] Burgess S Butterworth A Thompson SG. Mendelian randomization analysis with multiple genetic variants using summarized data. Genet Epidemiol 2013;37:658–65. 24114802 10.1002/gepi.21758 PMC4377079

[39] Pierce BL Burgess S. Efficient design for Mendelian randomization studies: subsample and 2-sample instrumental variable estimators. Am J Epidemiol 2013;178:1177–84. 23863760 10.1093/aje/kwt084 PMC3783091

[40] Zhu Z Zheng Z Zhang F. Causal associations between risk factors and common diseases inferred from GWAS summary data. Nat Commun 2018;9:224. 29335400 10.1038/s41467-017-02317-2 PMC5768719

[41] Xue A Zhu Z Wang H. Unravelling the complex causal effects of substance use behaviours on common diseases. Commun Med (Lond) 2024;4:43. 38472333 10.1038/s43856-024-00473-3 PMC10933313

[42] Bowden J Davey Smith G Burgess S. Mendelian randomization with invalid instruments: effect estimation and bias detection through Egger regression. Int J Epidemiol 2015;44:512–25. 26050253 10.1093/ije/dyv080 PMC4469799

[43] Chen Z Guo Y Sun H. Exploration of the causal associations between circulating inflammatory proteins, immune cells, and neuromyelitis optica spectrum disorder: a bidirectional Mendelian randomization study and mediation analysis. Front Aging Neurosci 2024;16:1394738. 38737586 10.3389/fnagi.2024.1394738 PMC11088236

[44] Zhang T Cao Y Zhao J Yao J Liu G. Assessing the causal effect of genetically predicted metabolites and metabolic pathways on stroke. J Transl Med 2023;21:822. 37978512 10.1186/s12967-023-04677-4 PMC10655369

[45] Barbeira AN Dickinson SP Bonazzola R. Exploring the phenotypic consequences of tissue specific gene expression variation inferred from GWAS summary statistics. Nat Commun 2018;9:1825. 29739930 10.1038/s41467-018-03621-1 PMC5940825

[46] Urbut SM Wang G Carbonetto P Stephens M. Flexible statistical methods for estimating and testing effects in genomic studies with multiple conditions. Nat Genet 2019;51:187–95. 30478440 10.1038/s41588-018-0268-8 PMC6309609

[47] Backman JD Li AH Marcketta A. Exome sequencing and analysis of 454,787 UK Biobank participants. Nature 2021;599:628–34. 34662886 10.1038/s41586-021-04103-z PMC8596853

[48] Weissbrod O Hormozdiari F Benner C. Functionally informed fine-mapping and polygenic localization of complex trait heritability. Nat Genet 2020;52:1355–63. 33199916 10.1038/s41588-020-00735-5 PMC7710571

[49] Jain P Miller-Fleming T Topaloudi A. Polygenic risk score-based phenome-wide association study identifies novel associations for Tourette syndrome. Transl Psychiatry 2023;13:69. 36823209 10.1038/s41398-023-02341-5 PMC9950421

[50] Cheng Y Wu S Chen S Wu Y. Association of body mass index combined with triglyceride-glucose index in cardiovascular disease risk: a prospective cohort study. Sci Rep 2025;15:17687. 40399481 10.1038/s41598-025-02342-y PMC12095778

[51] Wang L Cong HL Zhang JX. Triglyceride-glucose index predicts adverse cardiovascular events in patients with diabetes and acute coronary syndrome. Cardiovasc Diabetol 2020;19:80. 32534586 10.1186/s12933-020-01054-z PMC7293784

[52] Wang B Li L Tang Y Ran X. Joint association of triglyceride glucose index (TyG) and body roundness index (BRI) with stroke incidence: a national cohort study. Cardiovasc Diabetol 2025;24:164. 40241070 10.1186/s12933-025-02724-6 PMC12004739

[53] Wu L Bao X Xu J Ma L Kang L Zhang R. The triglyceride-glucose index positively associates with the prevalence and severity of coronary heart disease in patients among hypertension. Sci Rep 2025;15:19571. 40467641 10.1038/s41598-025-03948-y PMC12137601

[54] Lu L Chen Y Liu B. Association between cumulative changes of the triglyceride glucose index and incidence of stroke in a population with cardiovascular-kidney-metabolic syndrome stage 0–3: a nationwide prospective cohort study. Cardiovasc Diabetol 2025;24:202. 40355933 10.1186/s12933-025-02754-0 PMC12070779

[55] Gao X Chen T Zhou F. The association between different insulin resistance surrogates and all-cause mortality and cardiovascular mortality in patients with metabolic dysfunction-associated steatotic liver disease. Cardiovasc Diabetol 2025;24:200. 40346671 10.1186/s12933-025-02758-w PMC12065324

[56] Xu Z Yan X Li D Huang X. Triglyceride glucose index as a biomarker for heart failure risk in H-type hypertension patients. Sci Rep 2025;15:4828. 39924562 10.1038/s41598-025-89211-w PMC11808078

[57] Smemo S Tena JJ Kim KH. Obesity-associated variants within FTO form long-range functional connections with IRX3. Nature 2014;507:371–75. 24646999 10.1038/nature13138 PMC4113484

[58] Bosma M Gerling M Pasto J. FNDC4 acts as an anti-inflammatory factor on macrophages and improves colitis in mice. Nat Commun 2016;7:11314. 27066907 10.1038/ncomms11314 PMC4832079

[59] Larter CZ Chitturi S Heydet D Farrell GC. A fresh look at NASH pathogenesis. Part 1: the metabolic movers. J Gastroenterol Hepatol 2010;25:672–90. 20492324 10.1111/j.1440-1746.2010.06253.x

[60] Santoro N Zhang CK Zhao H. Variant in the glucokinase regulatory protein (GCKR) gene is associated with fatty liver in obese children and adolescents. Hepatology 2012;55:781–89. 22105854 10.1002/hep.24806 PMC3288435

[61] Bryan B Kumar V Stafford LJ Cai Y Wu G Liu M. GEFT, a Rho family guanine nucleotide exchange factor, regulates neurite outgrowth and dendritic spine formation. J Biol Chem 2004;279:45824–32. 15322108 10.1074/jbc.M406216200

[62] Allred N Raut C Chen Y. 348-OR: multiancestry Whole Genome Sequencing (WGS) meta-analysis to identify loci associated with imaging-measured hepatic steatosis. Diabetes 2024;73:348–OR.38377447

[63] Birker-Robaczewska M Boucher M Ranieri G. The novel lysophosphatidic acid receptor 1-selective antagonist, ACT-1016-0707, has unique binding properties that translate into effective antifibrotic and anti-inflammatory activity in different models of pulmonary fibrosis. J Pharmacol Exp Ther 2025;392:103396. 40073729 10.1016/j.jpet.2025.103396

[64] Kaffe E Magkrioti C Aidinis V. Deregulated lysophosphatidic acid metabolism and signaling in liver cancer. Cancers (Basel) 2019;11:1626. 31652837 10.3390/cancers11111626 PMC6893780

[65] Kisseleva T Brenner D. Molecular and cellular mechanisms of liver fibrosis and its regression. Nat Rev Gastroenterol Hepatol 2021;18:151–66. 33128017 10.1038/s41575-020-00372-7

[66] Piras IS Don J Schork NJ, and DiStefano JK. Potential causal links between genetic variants in SAMM50, SUGP1, MAU2, and GATAD2A and liver fat in individuals with normal weight. medRxiv [Preprint] 2024. doi:10.1101/2024.11.05.24316758

[67] Deng G-X Yin R-X Guan Y-Z. Association of the NCAN-TM6SF2-CILP2-PBX4-SUGP1-MAU2 SNPs and gene-gene and gene-environment interactions with serum lipid levels. Aging-US 2020;12:11893–913. 10.18632/aging.103361 PMC7343441 32568739

[68] Liu C Sekine S Ito K. Assessment of mitochondrial dysfunction-related, drug-induced hepatotoxicity in primary rat hepatocytes. Toxicol Appl Pharmacol 2016;302:23–30. 27095095 10.1016/j.taap.2016.04.010

[69] Ding N Wang K Jiang H. AGK regulates the progression to NASH by affecting mitochondria complex I function. Theranostics 2022; 12:3237–50. 35547757 10.7150/thno.69826 PMC9065199

[70] Eslam M Mangia A Berg T. Diverse impacts of the rs58542926 E167K variant in TM6SF2 on viral and metabolic liver disease phenotypes. Hepatology 2016;64:34–46. 26822232 10.1002/hep.28475

[71] Dongiovanni P Petta S Maglio C. Transmembrane 6 superfamily member 2 gene variant disentangles nonalcoholic steatohepatitis from cardiovascular disease. Hepatology 2015;61:506–14. 25251399 10.1002/hep.27490

[72] Potthoff MJ Arnold MA McAnally J Richardson JA Bassel-Duby R Olson EN. Regulation of skeletal muscle sarcomere integrity and postnatal muscle function by Mef2c. Mol Cell Biol 2007;27:8143–51. 17875930 10.1128/MCB.01187-07 PMC2169182

[73] El Jamal SM Grada Z El Dinali MH. MEF2B is a member of the BCL6 gene transcriptional complex and induces its expression in diffuse large B-cell lymphoma of the germinal center B-cell-like type. Lab Invest 2019;99:539–50. 30446717 10.1038/s41374-018-0152-2

[74] Yen L Cao Z Wu X. Loss of Nrdp1 enhances ErbB2/ErbB3-dependent breast tumor cell growth. Cancer Res 2006;66:11279–86. 17145873 10.1158/0008-5472.CAN-06-2319

[75] Wu X Chen Z Chen Q Lin C Zheng X Yuan B. Nrdp1-mediated macrophage phenotypic regulation promotes functional recovery in mice with mild neurological impairment after intracerebral hemorrhage. Neuroscience 2024;545:16–30. 38431041 10.1016/j.neuroscience.2024.02.028

[76] Cassandri M Smirnov A Novelli F. Zinc-finger proteins in health and disease. Cell Death Discov 2017;3:17071. 29152378 10.1038/cddiscovery.2017.71 PMC5683310

[77] Ruan HB Dietrich MO Liu ZW. O-GlcNAc transferase enables AgRP neurons to suppress browning of white fat. Cell 2014;159:306–17. 25303527 10.1016/j.cell.2014.09.010 PMC4509746

[78] Cochran BJ Ong KL Manandhar B Rye KA. APOA1: a protein with multiple therapeutic functions. Curr Atheroscler Rep 2021;23:11. 33591433 10.1007/s11883-021-00906-7

[79] Chistiakov DA Orekhov AN Bobryshev YV. ApoA1 and ApoA1-specific self-antibodies in cardiovascular disease. Lab Invest 2016;96:708–18. 27183204 10.1038/labinvest.2016.56

[80] Taghibiglou C Rashid-Kolvear F Van Iderstine SC. Hepatic very low density lipoprotein-ApoB overproduction is associated with attenuated hepatic insulin signaling and overexpression of protein-tyrosine phosphatase 1B in a fructose-fed hamster model of insulin resistance. J Biol Chem 2002;277:793–803. 11598116 10.1074/jbc.M106737200

[81] Adiels M Taskinen MR Packard C. Overproduction of large VLDL particles is driven by increased liver fat content in man. Diabetologia 2006;49:755–65. 16463046 10.1007/s00125-005-0125-z

[82] Sparks JD Dong HH. FoxO1 and hepatic lipid metabolism. Curr Opin Lipidol 2009;20:217–26. 21037971 10.1097/MOL.0b013e32832b3f4c PMC2964835

[83] Hsu CC Kanter JE Kothari V Bornfeldt KE. Quartet of APOCs and the different roles they play in diabetes. Arterioscler Thromb Vasc Biol 2023;43:1124–33. 37226733 10.1161/ATVBAHA.122.318290 PMC10330679

[84] Allan CM Walker D Segrest JP Taylor JM. Identification and characterization of a new human gene (APOC4) in the apolipoprotein E, C-I, and C-II gene locus. Genomics 1995;28:291–300. 8530039 10.1006/geno.1995.1144

[85] Kamboh MI Aston CE, and Hamman RF. DNA sequence variation in human apolipoprotein C4 gene and its effect on plasma lipid profile. Atherosclerosis 2000;152:193–201. 10996355 10.1016/s0021-9150(99)00459-1

[86] Villar D Berthelot C Aldridge S. Enhancer evolution across 20 mammalian species. Cell 2015;160:554–66. 25635462 10.1016/j.cell.2015.01.006 PMC4313353

[87] Lee EY Yang HK Lee J. Triglyceride glucose index, a marker of insulin resistance, is associated with coronary artery stenosis in asymptomatic subjects with type 2 diabetes. Lipids Health Dis 2016; 15:155. 27633375 10.1186/s12944-016-0324-2 PMC5024477

[88] Mouzaki M Comelli EM Arendt BM. Intestinal microbiota in patients with nonalcoholic fatty liver disease. Hepatology 2013;58:120–27. 23401313 10.1002/hep.26319

[89] Cai W Qiu T Hu W Fang T. Changes in the intestinal microbiota of individuals with non-alcoholic fatty liver disease based on sequencing: an updated systematic review and meta-analysis. PLoS One 2024;19:e0299946. 38547205 10.1371/journal.pone.0299946 PMC10977702

